# Supplementary material for: An intermediate Rb–E2F activity state safeguards proliferation commitment
Source: Nature. 2024 Jun 26;631(8020):424–31. doi: 10.1038/s41586-024-07554-2 (PMC11236703; doi:10.1038/s41586-024-07554-2)
Supplement: Supplementary file 1 — The selected regions shown in the main and Extended Data Figures are marked in the uncropped images by rectangular frames. [file 41586_2024_7554_MOESM1_ESM.pdf]

nature portfolio

https://doi.org/10.1038/s41586-024-07554-2

Supplementary information

An intermediate Rb–E2F activity state safeguards proliferation commitment

In the format provided by the authors and unedited

Supplementary Fig. 1. The selected regions shown in the Main and Extended Figures are marked in the uncropped images by rectangular frames.

Extended Fig. 5b. original Western blots showing the phosphorylation of the EGFR at Y1045 and the total EGFR concentration. Two exposures are shown, left and middle, and size markers as control on the right. The data was used as a control to show that the EGFR inhibitor, gefitinib, suppressed the phosphorylation of EGFR. Western blots of phospho-EGFR (p-Y1045) and total-EGFR. 1 of 3 biological replicates. Cells were treated with DMSO or EGFRi (5 μM gefitinib) 42 h after release with starvation media + EGF (20 ng/mL) + CDK4/6i (1 μM). Cells were lysed 4 h after DMSO or EGFRi treatment for western blots.

Fig. 4e. original Western blots for the analysis of the two Rb bands and the phosphorylation of 9 different Rb sites. Size markers always on left. Corresponding blots of total Rb on the right. Each of the sets (a, b and c) is showing the phosphorylation of different sites. The data was used to support that Rb is preferentially phosphorylated at T373 and S608 compared to other sites. 1 of 3 biological replicates. Cells were released with starvation media + EGF (20 ng/mL) + various drugs. Cells released with DMSO, CDK4/6i (1 μM), CDK4/6i (1 μM) + CDK2i (10 μM) were assayed 16, 24, 24 h after release, respectively. Starved cells were assayed before release. a, T252 and T356. b, S608, T373, and S795. c, S780 and S788. d, T826 and S807/S811.

Fig. 4f. original Western blots for the time course analysis of four of the Rb phosphorylation sites (as marked on the Blots). Size markers always on left and corresponding blots of the total Rb on the right. The data was used to support the single cell results that Rb is first phosphorylated at T373 and S608 before Rb hyperphosphorylation. 1 of 3 biological replicates. Cells were released with starvation media + EGF (20 ng/mL) + CDK4/6i (1 μM). Cells were assayed 0, 8, 16, 24, 48 h after release (from the leftmost lane to the 5th lane). Top left, T373. Top right, S608. Bottom left, S807/S811. Bottom right, T826.

Extended Data Fig. 5b

a Phospho-/total-EGFR

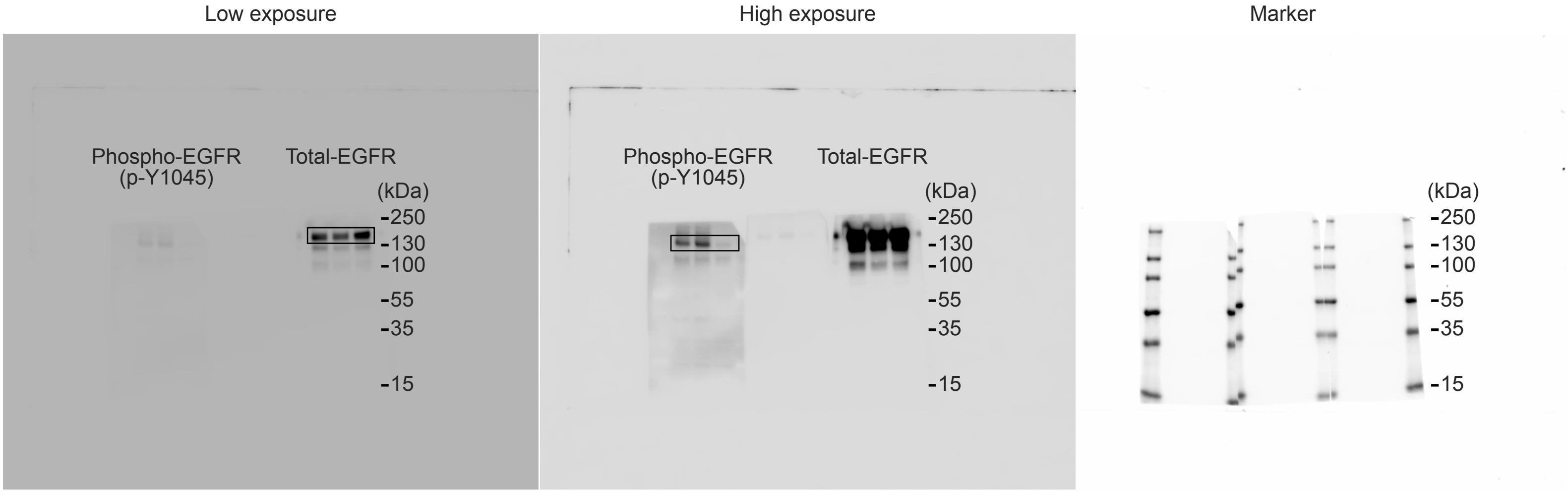

Fig. 4e

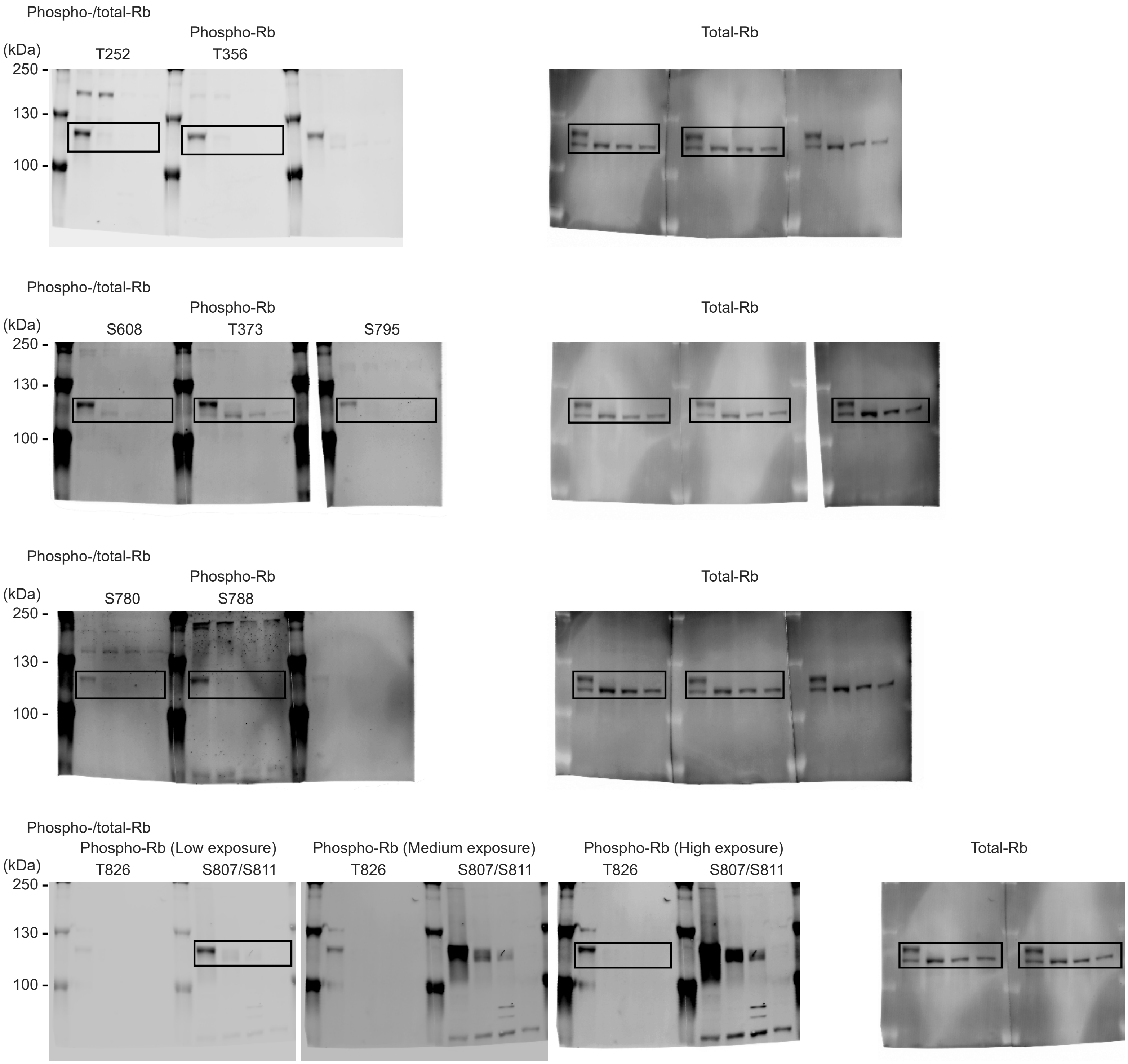
Phospho-/total-Rb
Phospho-Rb
Total-Rb
(kDa)
250
130
100
T252
T356
Phospho-/total-Rb
Phospho-Rb
Total-Rb
(kDa)
250
130
100
S608
T373
S795
Phospho-/total-Rb
Phospho-Rb
Total-Rb
(kDa)
250
130
100
S780
S788
Phospho-/total-Rb
Phospho-Rb (Low exposure)
Phospho-Rb (Medium exposure)
Phospho-Rb (High exposure)
Total-Rb
(kDa)
250
130
100
T826
S807/S811
T826
S807/S811
T826
S807/S811

Fig. 4f

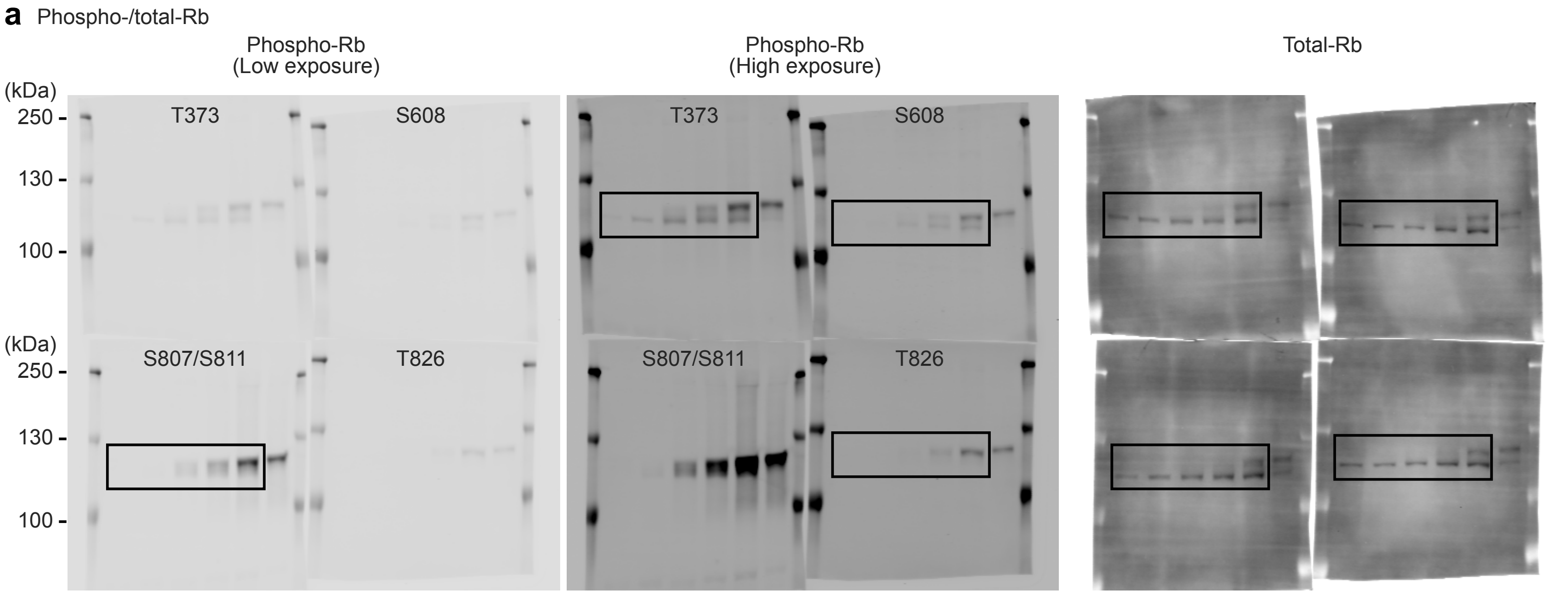
a Phospho-/total-Rb
Phospho-Rb
(Low exposure)
Phospho-Rb
(High exposure)
Total-Rb
(kDa)
250
130
100
T373
S608
S807/S811
T826
