# Supplementary material for: An intermediate Rb–E2F activity state safeguards proliferation commitment
Source: Nature. 2024 Jun 26;631(8020):424–31. doi: 10.1038/s41586-024-07554-2 (PMC11236703; doi:10.1038/s41586-024-07554-2)
Supplement: Supplementary file 2 — Reporting Summary [file 41586_2024_7554_MOESM2_ESM.pdf]

nature portfolio

Corresponding author(s): Tobias Meyer

Last updated by author(s): March 26, 2024

Reporting Summary

Nature Portfolio wishes to improve the reproducibility of the work that we publish. This form provides structure for consistency and transparency in reporting. For further information on Nature Portfolio policies, see our Editorial Policies and the Editorial Policy Checklist.

Statistics

For all statistical analyses, confirm that the following items are present in the figure legend, table legend, main text, or Methods section.

n/a	Confirmed	
☐	☒	The exact sample size (*n*) for each experimental group/condition, given as a discrete number and unit of measurement
☐	☒	A statement on whether measurements were taken from distinct samples or whether the same sample was measured repeatedly
☐	☒	The statistical test(s) used AND whether they are one- or two-sided<br>*Only common tests should be described solely by name; describe more complex techniques in the Methods section.*
☒	☐	A description of all covariates tested
☐	☒	A description of any assumptions or corrections, such as tests of normality and adjustment for multiple comparisons
☐	☒	A full description of the statistical parameters including central tendency (e.g. means) or other basic estimates (e.g. regression coefficient) AND variation (e.g. standard deviation) or associated estimates of uncertainty (e.g. confidence intervals)
☐	☒	For null hypothesis testing, the test statistic (e.g. *F*, *t*, *r*) with confidence intervals, effect sizes, degrees of freedom and *P* value noted<br>*Give P values as exact values whenever suitable.*
☒	☐	For Bayesian analysis, information on the choice of priors and Markov chain Monte Carlo settings
☒	☐	For hierarchical and complex designs, identification of the appropriate level for tests and full reporting of outcomes
☒	☐	Estimates of effect sizes (e.g. Cohen's *d*, Pearson's *r*), indicating how they were calculated

Our web collection on statistics for biologists contains articles on many of the points above.

Software and code

Policy information about availability of computer code

Data collection	MetaExpress version 6.1, a software provided by Molecular Devices, was used for acquisition of microscopy images on ImageXpress Micro. NIS-Elements AR version 5.21.03, a software provided by Nikon, was used for acquisition of microscopy images on Ti2-E.
Data analysis	A repository of Matlab code used for cell segmentation, tracking, and analysis is cited in the methods section.

For manuscripts utilizing custom algorithms or software that are central to the research but not yet described in published literature, software must be made available to editors and reviewers. We strongly encourage code deposition in a community repository (e.g. GitHub). See the Nature Portfolio guidelines for submitting code & software for further information.

Data

Policy information about availability of data

All manuscripts must include a data availability statement. This statement should provide the following information, where applicable:

- Accession codes, unique identifiers, or web links for publicly available datasets
- A description of any restrictions on data availability
- For clinical datasets or third party data, please ensure that the statement adheres to our policy

Raw imaging data acquired during this study have not been deposited in a public repository due to storage limitations but are available from the lead contact upon request.

Research involving human participants, their data, or biological material

Policy information about studies with human participants or human data. See also policy information about sex, gender (identity/presentation), and sexual orientation and race, ethnicity and racism.

Reporting on sex and gender	*Use the terms sex (biological attribute) and gender (shaped by social and cultural circumstances) carefully in order to avoid confusing both terms. Indicate if findings apply to only one sex or gender; describe whether sex and gender were considered in study design; whether sex and/or gender was determined based on self-reporting or assigned and methods used. Provide in the source data disaggregated sex and gender data, where this information has been collected, and if consent has been obtained for sharing of individual-level data; provide overall numbers in this Reporting Summary. Please state if this information has not been collected. Report sex- and gender-based analyses where performed, justify reasons for lack of sex- and gender-based analysis.*
Reporting on race, ethnicity, or other socially relevant groupings	*Please specify the socially constructed or socially relevant categorization variable(s) used in your manuscript and explain why they were used. Please note that such variables should not be used as proxies for other socially constructed/relevant variables (for example, race or ethnicity should not be used as a proxy for socioeconomic status). Provide clear definitions of the relevant terms used, how they were provided (by the participants/respondents, the researchers, or third parties), and the method(s) used to classify people into the different categories (e.g. self-report, census or administrative data, social media data, etc.) Please provide details about how you controlled for confounding variables in your analyses.*
Population characteristics	*Describe the covariate-relevant population characteristics of the human research participants (e.g. age, genotypic information, past and current diagnosis and treatment categories). If you filled out the behavioural & social sciences study design questions and have nothing to add here, write "See above."*
Recruitment	*Describe how participants were recruited. Outline any potential self-selection bias or other biases that may be present and how these are likely to impact results.*
Ethics oversight	*Identify the organization(s) that approved the study protocol.*

Note that full information on the approval of the study protocol must also be provided in the manuscript.

Field-specific reporting

Please select the one below that is the best fit for your research. If you are not sure, read the appropriate sections before making your selection.

☒ Life sciences ☐ Behavioural & social sciences ☐ Ecological, evolutionary & environmental sciences

For a reference copy of the document with all sections, see nature.com/documents/nr-reporting-summary-flat.pdf

Life sciences study design

All studies must disclose on these points even when the disclosure is negative.

Sample size	No sample-size calculation was performed. Sample size was determined to be adequate based on the magnitude and consistency of the measurable differences between groups. All experiments were repeated on multiple days to ensure reproducibility. For experiments where significance was calculated, n of at least 3 was predetermined to enable adequate statistical testing.
Data exclusions	Any failed experiments where cells were at suboptimal confluency, CO2, or temperature were excluded. For live-cell imaging analysis, cells were excluded if they could not be distinguished from a neighboring cell or were not tracked for a significant period of time in the experiment.
Replication	Attempts at replication were successful, and all experiments were repeated on multiple days. Number of times experiments were repeated can be found in the figure legends. Experiments where significance were derived have at least n=3.
Randomization	In cell line experiments, no randomization was required because the experimental cells came from the same source and were tested identically with the variable treatment. Cell lines were used for the entirety of this study; no other sample type that would require randomization was used in this study.
Blinding	Blinding was not done. The same automated analysis pipeline was applied to all conditions. Blinding was not considered based upon experience and similar published studies.

Reporting for specific materials, systems and methods

We require information from authors about some types of materials, experimental systems and methods used in many studies. Here, indicate whether each material, system or method listed is relevant to your study. If you are not sure if a list item applies to your research, read the appropriate section before selecting a response.

Materials & experimental systems

n/a	Involved in the study
☐	☒ Antibodies
☐	☒ Eukaryotic cell lines
☒	☐ Palaeontology and archaeology
☒	☐ Animals and other organisms
☒	☐ Clinical data
☒	☐ Dual use research of concern
☒	☐ Plants

Methods

n/a	Involved in the study
☐	☒ ChIP-seq
☒	☐ Flow cytometry
☒	☐ MRI-based neuroimaging

Antibodies

Antibodies used	All antibody catalog numbers are provided in the methods section.
Validation	Antibodies validated through siRNA knockdown and cellular localization using immunofluorescence: mouse anti-Rb antibody (BD Cat# 554136), rabbit anti-phospho-Rb (S807/S811) antibody (Cell Signaling Technology Cat# 8516), rabbit anti-p21 antibody (Cell Signaling Technology Cat# 2947), mouse anti-cyclin E antibody (Santa Cruz Cat# sc-247), rabbit anti-c-Myc antibody (Cell Signaling Technology #5605), rabbit anti-53BP1 antibody (Cell Signaling Technology Cat# 4937)<br><br>Antibodies validated through overexpression using immunofluorescence: rabbit anti-phospho-Rb (T373) antibody (Abcam Cat# ab52975), rabbit anti-phospho-Rb (S608) antibody (Cell Signaling Technology Cat# 2181), rabbit anti-HA tag antibody (Cell Signaling Technology Cat# 3724), mouse anti-HA tag antibody (BioLegend Cat# 901501)<br><br>Antibodies validated through band size using Western blots: mouse anti-Rb antibody (Cell Signaling Technology Cat# 9309), all anti-phospho-Rb antibodies were further validated with CDK4/6 inhibitor treatment.<br><br>Statements on manufacturer's website or comments about citations where relevant:<br>mouse anti-Rb antibody (BD Cat# 554136): "G3-245 was made using a Trp-E-Rb fusion protein as immunogen and recognizes an epitope between amino acids 332-344 (DARLFDHDKTLQ) of the human retinoblastoma protein (pp110-114 Rb)."<br><br>mouse anti-Rb antibody (Cell Signaling Technology Cat# 9309): "Rb (4H1) Mouse mAb detects endogenous levels of total Rb protein. The antibody does not cross-react with the Rb homologues p107 or p130, or with other proteins."<br><br>rabbit anti-phospho-Rb (S807/S811) antibody (Cell Signaling Technology Cat# 8516): "Phospho-Rb (Ser807/811) (D20B12) XP® Rabbit mAb recognizes endogenous levels of Rb protein only when phosphorylated at Ser807, Ser811, or at both sites. This antibody does not cross-react with Rb phosphorylated at Ser608."<br><br>rabbit anti-p21 antibody (Cell Signaling Technology Cat# 2947): "p21 Waf1/Cip1 (12D1) Rabbit mAb detects endogenous levels of total p21 protein. The antibody does not cross-react with other CDK inhibitors."

Eukaryotic cell lines

Policy information about [cell lines and Sex and Gender in Research]

Cell line source(s)	MCF-10A, RPE-1, BJ-5ta, U2OS cells were acquired directly from ATCC. Lenti-X 293T cells were acquired directly from Takara Bio.
Authentication	MCF-10A cells were validated by RNA-seq. Other cell lines were not validated.
Mycoplasma contamination	All cell lines tested negative for mycoplasma contamination. Our laboratory routlinely tests for myoplasma by a PCR test and by Hoechst 33342 DNA staining.
Commonly misidentified lines (See ICLAC register)	No commonly misidentified lines were used in this study.

Plants

Seed stocks

Report on the source of all seed stocks or other plant material used. If applicable, state the seed stock centre and catalogue number. If plant specimens were collected from the field, describe the collection location, date and sampling procedures.

Novel plant genotypes

Describe the methods by which all novel plant genotypes were produced. This includes those generated by transgenic approaches, gene editing, chemical/radiation-based mutagenesis and hybridization. For transgenic lines, describe the transformation method, the number of independent lines analyzed and the generation upon which experiments were performed. For gene-edited lines, describe the editor used, the endogenous sequence targeted for editing, the targeting guide RNA sequence (if applicable) and how the editor was applied.

Authentication

Describe any authentication procedures for each seed stock used or novel genotype generated. Describe any experiments used to assess the effect of a mutation and, where applicable, how potential secondary effects (e.g. second site T-DNA insertions, mosiacism, off-target gene editing) were examined.

ChIP-seq

Data deposition

☒ Confirm that both raw and final processed data have been deposited in a public database such as GEO.

☐ Confirm that you have deposited or provided access to graph files (e.g. BED files) for the called peaks.

Data access links
May remain private before publication.

Publicly available datasets downloaded from ENCODE are presented in our study.

Files in database submission

E2F1 in HepG2: https://www.encodeproject.org/experiments/ENCSR717ZZW/
E2F1 in MCF-7: https://www.encodeproject.org/experiments/ENCSR000EWX/
E2F2 in HepG2: https://www.encodeproject.org/experiments/ENCSR013RNH/
E2F3 in K562: https://www.encodeproject.org/experiments/ENCSR036QIR/
E2F4 in HepG2: https://www.encodeproject.org/experiments/ENCSR924LSO/
E2F4 in MCF-7: https://www.encodeproject.org/experiments/ENCSR505NMN/
E2F5 in HepG2: https://www.encodeproject.org/experiments/ENCSR486JYI/
E2F6 in A549: https://www.encodeproject.org/experiments/ENCSR000BTC/
E2F7 in K562: https://www.encodeproject.org/experiments/ENCSR171CAY/
E2F8 in HepG2: https://www.encodeproject.org/experiments/ENCSR634GEO/
E2F8 in MCF-7: https://www.encodeproject.org/experiments/ENCSR897ZXU/

Genome browser session
(e.g. UCSC)

Provide a link to an anonymized genome browser session for "Initial submission" and "Revised version" documents only, to enable peer review. Write "no longer applicable" for "Final submission" documents.

Methodology

Replicates

Describe the experimental replicates, specifying number, type and replicate agreement.

Sequencing depth

Describe the sequencing depth for each experiment, providing the total number of reads, uniquely mapped reads, length of reads and whether they were paired- or single-end.

Antibodies

Describe the antibodies used for the ChIP-seq experiments; as applicable, provide supplier name, catalog number, clone name, and lot number.

Peak calling parameters

Specify the command line program and parameters used for read mapping and peak calling, including the ChIP, control and index files used.

Data quality

Describe the methods used to ensure data quality in full detail, including how many peaks are at FDR 5% and above 5-fold enrichment.

Software

Describe the software used to collect and analyze the ChIP-seq data. For custom code that has been deposited into a community repository, provide accession details.
